# Functional exploration of *CCNE1* splicing forms as a possible link to bladder cancer susceptibility

**DOI:** 10.1186/gb-2011-12-s1-p7

**Published:** 2011-09-19

**Authors:** Indu Kohaar, Alexandra Scott-Johnson, Yi-Ping Fu, Patricia Porter-Gill, Ludmila Prokunina-Olsson

**Affiliations:** 1 Laboratory of Translational Genomics, Division of Cancer Epidemiology and Genetics, National Cancer Institute, National Institutes of Health, Bethesda, MD 20892, USA

## Background

A recent genome-wide association study (GWAS) identified a single nucleotide polymorphism, rs8102137, located 6 kb upstream of the cyclin E1 gene (*CCNE1*) on chromosome 19q12, as a risk factor for bladder cancer (odds ratio (OR) = 1.13, *P* = 1.7 × 10^–11^) [1]. *CCNE1* encodes a cell cycle protein that regulates cyclin-dependent kinases and is therefore an important cancer susceptibility gene.

## Methods

This study used 42 bladder tumor samples and 41 normal bladder tissue samples (24 matched normal-tumor pairs), HeLa cells and several prostate and bladder cancer cell lines. Genotyping of rs8102137 in DNA and rs7257694 in both DNA and cDNA samples was performed using an allelic discrimination genotyping assay. TaqMan and SYBR Green assays were used to measure the expression of the different *CCNE1* isoforms. The *CCNE1* isoforms were cloned into a pFC14A (HaloTag) CMV Flexi Vector. Protein expression of *CCNE1* isoforms in normal and tumor bladder tissues and transfected cells was analyzed by western blotting. Subcellular localization of recombinant *CCNE1* splicing forms was analyzed by confocal microscopy.

## Results

*CCNE1* mRNA was expressed at a higher level in bladder tumors (*n* = 42) than in adjacent normal bladder tissue samples (*n* = 41, 3.7fold, *P* = 2.7 × 10^–12^). However, no association was found between mRNA expression level and the genotype of rs8102137. We observed strong allelic expression imbalance for a synonymous coding variation located in the last exon (rs7257694, Ser390Ser), which is in high linkage disequilibrium with rs8102137 (normal bladder tissue samples, *n* = 41, D′ = 1.0, *r*^2^ = 0.815; HapMap CEU samples, *n* = 60, D′ = 0.95, *r*^2^ = 0.68). In normal and tumor tissue samples heterozygous for both single nucleotide polymorphisms, the risk variant of rs8102137 was associated with lower expression of allele T of rs7257694 (normal samples, *P* = 2.2 × 10^–4^; tumor samples, *P* = 1.11 × 10^–10^). Western blotting analysis of bladder tissue and prostate cell line lysates revealed that the allelic expression imbalance is likely to be related to two CCNE1 protein isoforms that showed a differential pattern of expression dependent on the rs8102137 and rs7257694 genotype. We have cloned the alternative splicing forms of *CCNE1* and are currently evaluating their functional relevance.

## Conclusions

Our results suggest that bladder-cancer-associated genetic variants of the *CCNE1* gene might contribute to altered cell cycle regulation, owing to differential mRNA splicing producing different protein isoforms of CCNE1.

## References

[B1] Rothman N Garcia-Closas M Chatterjee N Malats N Wu X Figueroa JD Real FX Van Den Berg D Matullo G Baris D Thun M Kiemeney LA Vineis P De Vivo I Albanes D Purdue MP Rafnar T Hildebrandt MA Kiltie AE Cussenot O Golka K Kumar R Taylor JA Mayordomo JI Jacobs KB Kogevinas M Hutchinson A Wang Z Fu YP Prokunina-Olsson L A multi-stage genome-wide association study of bladder cancer identifies multiple susceptibility loci Nat Genet 2010 42 978 984 10.1038/ng.687 20972438 PMC3049891

